# Pupillometry and brain-wide c-Fos mapping uncover multimodal mirror emotional contagion related networks of mice

**DOI:** 10.1016/j.isci.2026.114827

**Published:** 2026-01-29

**Authors:** Matteo Caldarelli, Stefano Zucca, Aurelia Viglione, Alessandra Stella, Rida Nisar, Giulia Sagona, Ester M. Papini, Fabio Carrara, Serena Bovetti, Raffaele M. Mazziotti, Tommaso Pizzorusso

**Affiliations:** 1 BIO@SNS Lab, Scuola Normale Superiore, Pisa, Italy; 2 Department of Life Sciences and Systems Biology, Neuroscience Institute Cavalieri Ottolenghi, University of Torino, Orbassano, Italy; 3 Institute of Neuroscience, National Research Council, Pisa, Italy; 4 Institute of Information Science and Technologies, National Research Council, Pisa, Italy Institute of Neuroscience, National Research Council, Pisa, Italy; 5 Department of Neuroscience, Psychology, Pharmacology, and Child Health, University of Florence, Florence, Italy; 6 Department of Developmental Neuroscience, IRCCS Stella Maris Foundation, Pisa, Italy

**Keywords:** neuroscience, behavioral neuroscience, molecular neuroscience

## Abstract

Emotional contagion (ECo) represents a fundamental form of empathy. In this study, we used pupillometry to quantify ECo by assessing pupil responses of a mouse watching another mouse receive a tail shock. Pupil dilation effectively measured both direct and vicarious emotional response thresholds at the individual level through psychometric curve analysis. The pupillary ECo response diminished when the observer could not see the demonstrator, suggesting a multisensory process involving vision. Viewing videos of tail-shocked mice elicited a pupil response in the observer. Brain-wide c-Fos mapping revealed a broad network of 88 brain regions activated during ECo, with all areas activated in the demonstrator also engaged in the observer. Additionally, in some brain regions, correlated activation was detected between each observer-demonstrator pair, indicating that ECo promotes a shared neural state. These findings advance our understanding of the neural basis of shared emotions, with implications for analyzing neuropsychiatric disorder models.

## Highlights

• Pupillometry tracks direct and vicarious emotional responses
• Visual cues alone can trigger emotional contagion
• Emotional contagion recruits a distributed whole-brain network
• Observers and demonstrators show coupled pupil and neural activation

## Graphical abstract

**Figure:**
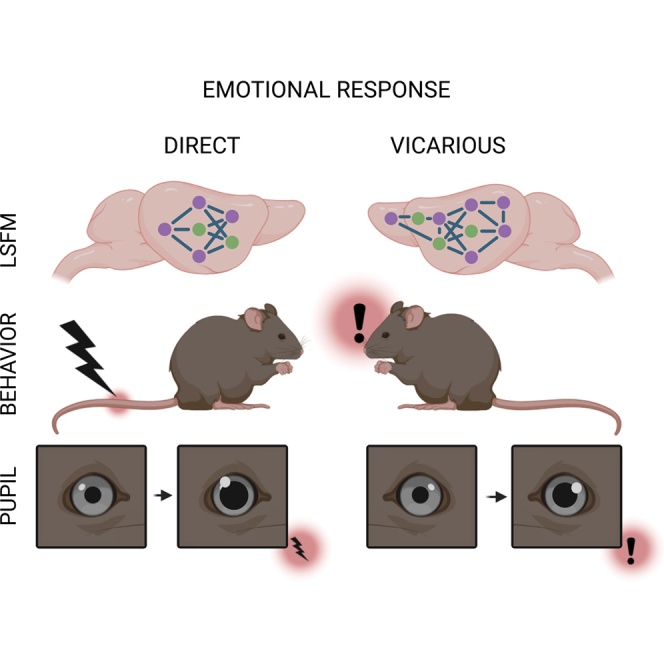

## Introduction

Empathy refers to the sharing of emotional states between individuals.^1^ This multifaceted concept spans from basic behavioral reactions to complex constructs like prosocial behavior, perspective-taking, and theory of mind.^2^ Emotional contagion (ECo), one of the most simple forms of empathy, is an automatic emotional response to others emotions.^3^ Extensive research has been conducted on ECo in humans^3^^,^^4^ and animals,^5^ revealing its presence in most of tested mammals and even in other groups such as in birds, like corvids,^6^^,^^7^ fish,^8^^,^^9^ and in much simpler animals such as isopods.^10^ Several paradigms have been used to study ECo in rodents,^11^^,^^12^^,^^13^ typically involving an observer (OBS) and a demonstrator (DEM).^14^ In the *Vicarious freezing* test, the OBS increases freezing behavior in response to the negative emotions experienced by the DEM. In the *Vicarious Learning* paradigm, the OBS learns new associations between stimuli by perceiving the emotional reactions of the DEM. These paradigms illustrate how ECo enables animals to avoid threats without direct exposure.^11^

ECo leads to a change of internal emotional state.^11^ Internal states involve dynamic variations in physiological and behavioral variables in response to stimuli. These pleiotropic effects consist of changes across different systems and occur in a coordinated manner. The extent of these fluctuations is proportional to the intensity of the stimulus.^15^ Therefore, ECo is expected to share these characteristics, manifesting as widespread and concurrent changes across multiple physiological variables, with the magnitude of these responses corresponding to the emotional intensity evoked by the stimulus. The triggering of the pleiotropic effects involves modifications in the activity of the autonomic nervous system, which in turn are reflected on pupil size.^16^^,^^17^ For this reason pupillometry can be potentially considered as a window on the emotional system.^16^^,^^17^^,^^18^^,^^19^^,^^20^ Pupillary activity can also address variations in the brain state not associated with noticeable changes in overt behavior.^21^^,^^22^^,^^23^^,^^24^ This indicates that pupillometry has high sensitivity to small and transient arousal fluctuations and it can detect both overt and covert behavioral phenomena. For these reasons, we evaluated the effectiveness of pupillometry in quantifying direct emotional response (DER) elicited by aversive stimulation, and vicarious emotional response (VER) elicited by witnessing the DER expressed by a conspecific. Considering that previous work demonstrated that a pre-exposure to the same stimulus potentiate ECo,^25^^,^^26^ mice undergoing VER were previously exposed to DER. We also analyzed pupillometry data separately for each sex, as sex differences might affect emotional responses, a factor not well-explored due to limited studies on female mice.^27^

Research has identified various brain areas involved in ECo in rodents, with most studies highlighting the significant role of the cortico-limbic circuit^27^^,^^28^ and suggesting a significant overlap in neural activation between rodents and humans during ECo.^27^ c-Fos activation was observed in several areas of the mouse brain such as the anterior cingulate cortex (ACA), prelimbic cortex (PL), and infralimbic cortex (IL), along with subcortical structures like the nucleus accumbens (NAc), mediodorsal thalamus (MD), lateral habenula (LH), and the amygdalar complex.^29^^,^^30^^,^^31^^,^^32^^,^^33^^,^^34^^,^^35^ However, no studies have conducted a comprehensive analysis of brain-wide activation and network connectivity involved in ECo, nor have they explored how this activation relates to interactions between subjects in a dyad. In this work, we employed light-sheet microscopy to detect the areas activated during ECo, providing a detailed map of brain-wide activation. To further characterize this activation, we applied connectivity analysis, which identified anatomically associated modules and provided a systems-level perspective on circuit organization during ECo. Finally, we conducted a correlation analysis to determine if the activation patterns in the DEM are related to those in the OBS, offering new insights into how activation may be coordinated between subjects in a dyad.

## Results

### Pupil size and locomotor activity are quantitative indicators of emotional reactivity to direct aversive stimulation

**Figure 1:**
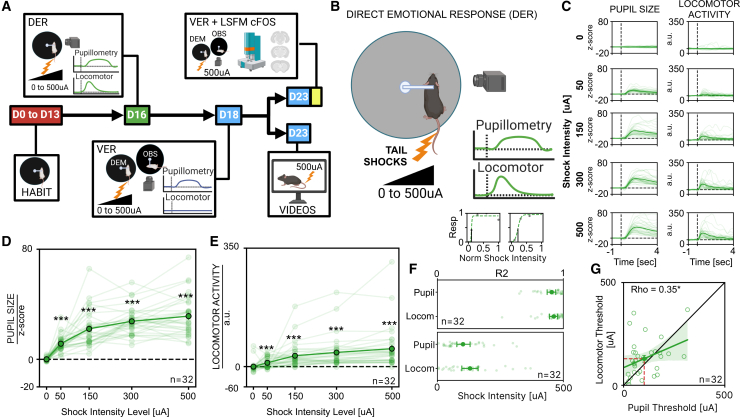
Experimental timeline and effects of DER on pupillary and locomotor activity in mice (A) Experimental timeline: All animals underwent the same longitudinal sequence, including habituation to head-fixation, DER, VER, and VER with occluder. On day 23, a subset of animals was assigned either to VER for c-Fos analysis, or to the video condition. (B) Sketch of the setup: head-fixed mice on a circular treadmill received tail shocks ranging from 0 to 500 μA while pupil size and locomotor activity were recorded simultaneously. (C) Grand average temporal profiles of pupil size (left) and locomotor activity (right) across stimulation intensities. Thin lines show individual animal averages; the grand average is their mean. (D) Average pupillary dilation as a function of shock amplitude. Thin lines represent individual mouse traces, thick lines indicate the average response across all animals (*n* = 32; Friedman test, χ^2^(4) = 99.8, *p* < 0.0001, BF_10_ ≥ 10^4^; followed by Wilcoxon tests for multiple comparisons). Asterisks indicate significance vs. 0 μA stimulation. (E) Average locomotor activity changes as a function of shock amplitude. Thin lines represent individual mouse traces, thick lines indicate the average response across all animals (*n* = 32; Friedman test, χ^2^(4) = 87.8, *p* < 0.0001, BF_10_ ≥ 10^4^; followed by Wilcoxon tests for multiple comparisons). Asterisks indicate significance vs. 0 μA stimulation. (F) Top: representative single-animal pupillary and locomotor responses fitted by psychometric curves. Solid gray circles represent normalized responses, and the dashed green lines indicate the fitted curves. Vertical gray lines mark the threshold level for each psychometric curve, representing the intensity value at 50% of the maximal response. Middle: goodness of psychometric curve fitting (R^2^) of the data. Lighter points represent single-animal values, solid points represent mean ± SEM (*n* = 32; paired *t* test, t(31) = −0.85, *p* = 0.40, Cohen’s d = 0.18, BF_10_ = 0.26). Bottom: comparison of response thresholds for DER pupil and DER locomotor. Lighter points represent single-animal thresholds, solid points represent mean ± SEM (*n* = 32; paired *t* test, t(31) = −1.57, *p* = 0.13, Cohen’s d = 0.32, BF_10_ = 0.57). (G) Correlation between locomotor and pupillary thresholds. Thick line indicates correlation line, the solid circle is the average correlation across all subjects ± SEM and shaded area is 95% CI (*n* = 32; Spearman correlation, *ρ* = 0.35, *p* = 0.047). OBS: observer, DEM: demonstrator. (∗: *p* < 0.05. ∗∗: *p* < 0.01, ∗∗∗: *p* < 0.001).

To assess whether pupillometry can detect shifts in internal states due to direct aversive stimulation, we developed a paradigm where head-fixed mice are subjected to tail shocks of varying intensities while simultaneously monitoring both pupillary and locomotor activity (Figure 1B). Tail shocks resulted in a significant transient increase in both pupil dilation and locomotor activity across all intensities relative to baseline (Figure 1C). Moreover, the magnitude of these responses scaled proportionally with the intensity of the stimulation (Figures 1D and 1E). The locomotor response to tail shock observed here contrasts with earlier findings of freezing behavior following aversive stimuli. This variation aligns with previous literature, which shows that whether a mouse flees or freezes in response to a threatening stimulus depends on environmental, situational, and biological factors.^36^^,^^37^^,^^38^ These findings indicate that pupillometry, alongside locomotor activity, provides a reliable marker of DERs to aversive stimuli, with both pupil dilation and locomotion reflecting the subjective experience of the stimulus.

To further investigate the capacity of pupillary and locomotor activity to profile individual sensitivity to aversive stimulation, we applied psychometric fitting to the responses of individual animals (Figures 1F and S1). This method allows us to quantify how each DER of the subject scales with stimulus intensity, providing parameters that describe the performance. The goodness-of-fit metric demonstrates that psychometric fitting accurately models both pupillary and locomotor response curves, with no significant differences in fit quality or average responsiveness thresholds between the two measures. No difference in DER threshold was present between male and female mice (Figures S2A, S2B, asnd S2D). We also found that pupillary and locomotor response thresholds are correlated within subjects, suggesting that both pupillary and locomotor activity can effectively quantify the emotional reactivity of the subject to aversive events (Figure 1G).

### Pupil size, but not locomotor activity, is a quantitative indicator of rapid vicarious emotional response

**Figure 2:**
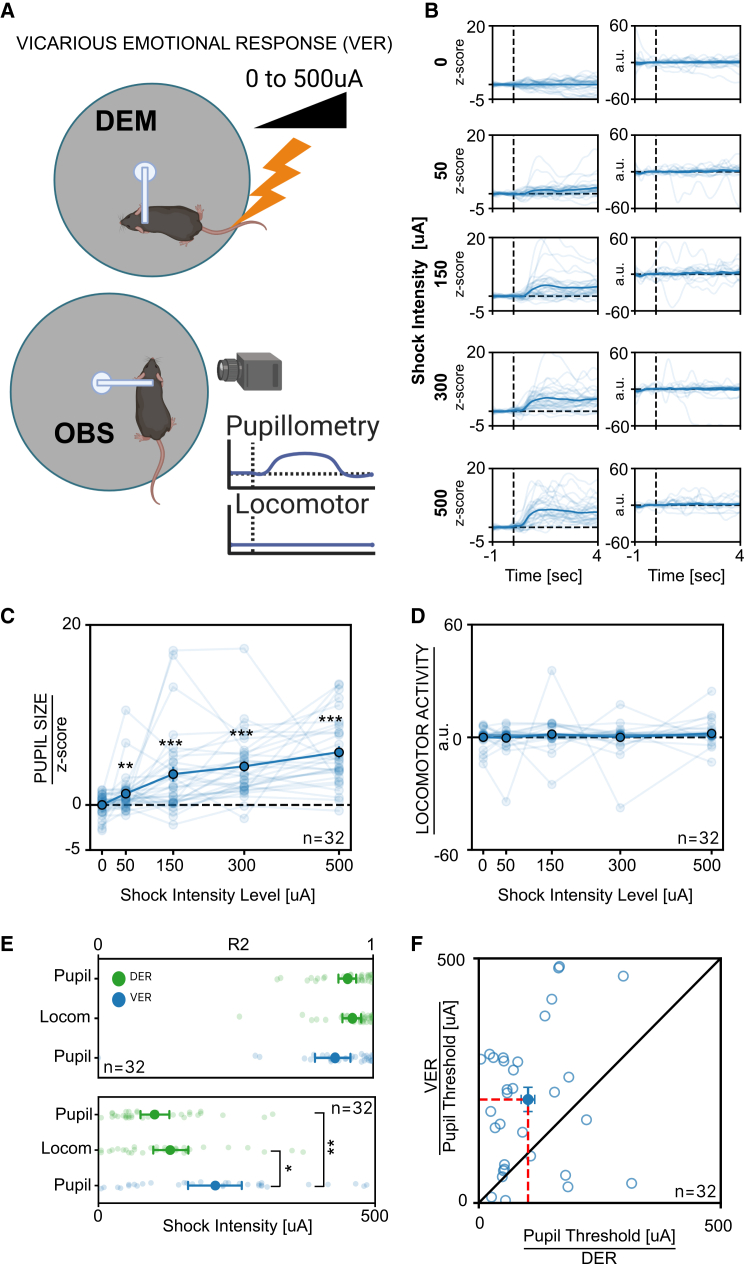
Effects of VER on pupillary and locomotor activity in mice (A) Sketch of the experimental setup: two head-fixed mice were placed on separate circular treadmills, allowing visual, olfactory, and auditory sensory interaction. Tail shocks ranging from 0 to 500 μA were applied to DEM, while pupil size and locomotor activity were recorded in OBS. (B) Grand average of the temporal profile of pupil size (left) and locomotor activity (right) of OBS at different stimulation intensities. The vertical dashed line marks the onset of the stimulus. Thin lines show individual animal averages; the grand average is their mean. (C) Average pupillary dilation of OBS as a function of the shock amplitude applied to DEM. Thin lines represent individual OBS averages, thick lines indicate the average response across all OBS (*n* = 32; Friedman test, χ^2^(4) = 67.6, *p* < 0.0001, BF_10_ ≥ 10^4^; followed by Wilcoxon tests for multiple comparisons). Asterisks indicate significance vs. 0 μA stimulation. (D) Average locomotor activity of OBS as a function of the shock amplitude applied to DEM. Thin lines represent individual OBS averages, the thick line represents the average response across all OBS (*n* = 32; Friedman test, χ^2^(4) = 5.52, *p* = 0.238, BF_10_ = 0.077). (E) Top: goodness of psychometric curve fitting for VER (pupillary) and DER (pupillary and locomotor activity). Lighter points represent single-animal values, solid points represent mean ± SEM (*n* = 32; Kruskal-Wallis test, H(2) = 2.84, *p* = 0.242, BF_10_ = 0.41). Bottom: comparison of response thresholds between VER (pupil) and DER (pupillary and locomotor activity) conditions. Light points represent single-animal thresholds, solid points represent mean ± SEM (*n* = 32; Kruskal-Wallis test, H(2) = 11.7, *p* = 0.003, BF_10_ = 97.8; followed by Mann-Whitney tests for multiple comparisons). (F) Correlation between OBS pupillary response thresholds during VER and DER conditions. Empty circles represent values for individual animals, the solid circle indicates the mean value across subjects ± SEM (*n* = 32; Spearman correlation, ρ = 0.075, *p* = 0.68). OBS: observer, DEM: demonstrator. (∗: *p* < 0.05. ∗∗: *p* < 0.01, ∗∗∗: *p* < 0.001).

**Figure 3:**
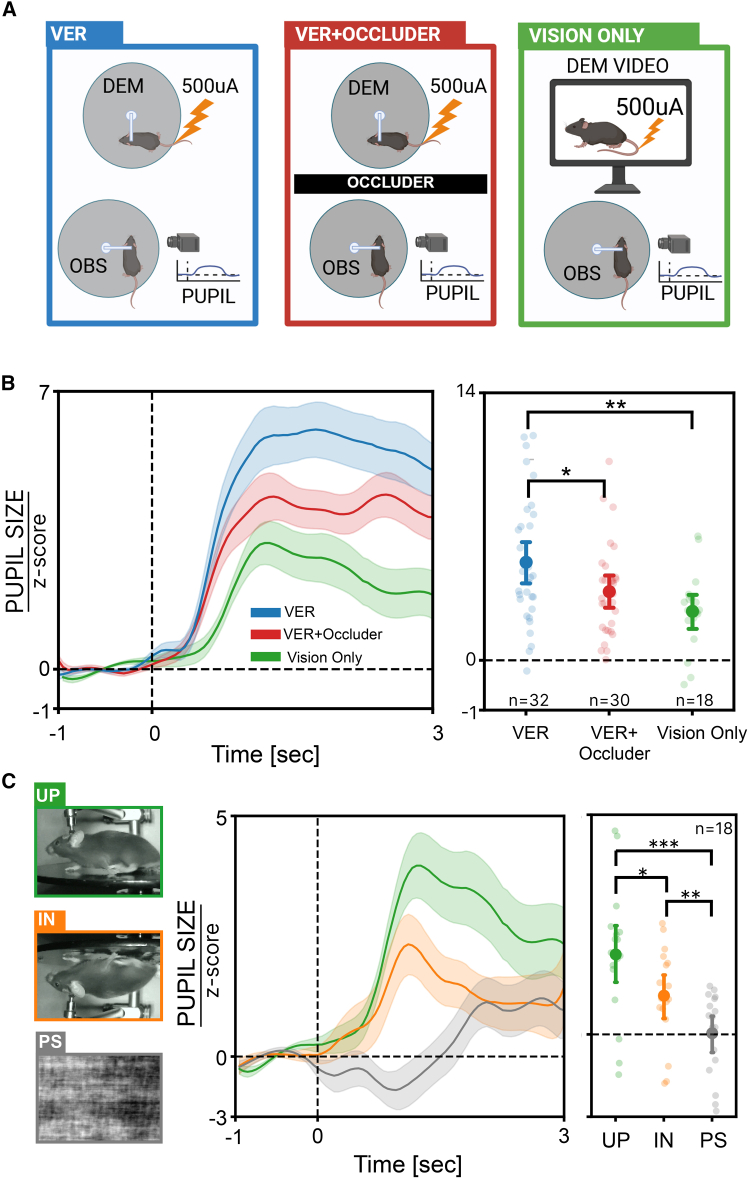
Multisensory integration underlies pupillary responses to VER (A) Sketch of the experimental setting. In the VER condition, OBS and DEM are in multisensory contact. In the VER+Occluder condition, a barrier is placed between OBS and DEM and shocks of 0 or 500 μA are delivered to DEM. In the vision only condition, OBS watches a video of DEM receiving either 0 or 500 μA shocks. (B) Left: time course of the pupillary responses of all OBS after 500 μA stimulation of DEM (or DEM videos) in the VER, VER+Occluder, and vision only conditions. The vertical dashed line indicates stimulus onset, and the shaded area around the solid lines represents SEM. Right: average pupillary dilation of all OBS to 500 μA stimulation in the VER, VER+Occluder, and vision only conditions. Solid circles represent the mean, and lighter circles represent single-animal data. Data are presented as mean ±95% confidence interval (n_VER_ = 32, n_VER+Occluder_ = 30, n_Vision Only_ = 18; one-way ANOVA, F = 5.39, *p* = 0.006, BF_10_ = 6.64; followed by post hoc tests for multiple comparisons). (C) Left: example frames from movies used for video administration. UP is a video of a mouse receiving the shock; PS is its phase-scrambled version; IN is the same video but vertically flipped. Center: time course of pupil responses of all observers after 500 μA stimulation of the DEM in the three video administration conditions. The vertical dashed line indicates stimulus onset, and the shaded area around the solid lines represents SEM. Right: average pupillary dilation of all observers to 500 μA stimulation in the UP, PS, and IN conditions. Solid points represent the mean, and lighter points represent single-animal data. Data are presented as mean ± SEM (*n* = 18; Friedman test, χ^2^(2) = 16.3, *p* = 2.8 × 10^−4^, BF_10_ ≥ 10^4^; followed by Wilcoxon tests for multiple comparisons). OBS: observer, DEM: demonstrator. (∗: *p* < 0.05. ∗∗: *p* < 0.01, ∗∗∗: *p* < 0.001).

To evaluate whether pupillary and locomotor activity can track ECo in the observing mouse, we assessed VER using two mice: an OBS and a DEM. Considering that pre-exposure to the same stimulus potentiate ECo,^25^^,^^26^ observers were previously exposed to DER. Both mice were head-fixed and maintained in multisensory contact, allowing for auditory, visual, and olfactory interaction (Figure 2A). The DEM received tail shocks of varying intensities, while the pupillary and locomotor activity of the OBS were recorded. We found that when the DEM received a tail shock, the OBS exhibited a significant pupil dilation, while no changes in locomotor activity were detected (Figures 2B–2D). Since VER could accumulate or interact over time we analyzed the data of the single VER trials during the 500 μA stimulation. Our results showed no significant changes in the pupillary response between the first and last repetition (Figure S3C). This demonstrates that an interaction effect from repeated stimulation is negligible within our experimental protocol. Overall, these findings demonstrate that pupil size measures can effectively track VER occurring in the OBS mouse. Furthermore, applying psychometric fitting to pupillary VER of individual observers revealed a good fit quality, comparable to that observed measuring DER (Figure 2E). This suggests that psychometric fitting of pupillary VER is a reliable method for assessing ECo thresholds at the single-subject level (Figure S1). No difference in VER threshold was present between male and female mice (Figures S2C and S2D). Notably, the VER threshold was significantly higher than the DER threshold (Figure 3E). The analysis of VER and DER thresholds in the same animal did not show a significant correlation between these measures, suggesting that the processes determining the sensitivity to DER and VER can be dissociated (Figure 2F).

### Visual input contributes to ECo

Previous research indicates that ECo is a multimodal process, with most studies focusing on the contribution of olfactory and auditory stimuli,^27^ however, little is known about the role of vision in ECo. To address this question, we implemented two modified versions of the VER experiment: the VER+Occluder condition, in which an opaque screen was positioned between the animals to obstruct the OBS view, aimed at determining whether ECo operates via non-visual sensory modalities; and the vision only condition, where the DEM was replaced by a video of a mouse undergoing DER, to assess the contribution of visual cues to ECo (Figure 3A). We exclusively used the maximum stimulus intensity of 500 μA, or videos featuring mice that received this maximal intensity. We found that the VER+Occluder condition evoked a significantly smaller pupil dilation as compared to the response to the VER condition (Figure 3B). This indicates that although vision is not strictly required to elicit a vicarious pupillary response, it significantly modulates its amplitude. This observation suggests that ECo processing undergoes multisensory enhancement, wherein the integration of multiple sensory inputs leads to a more pronounced response, amplifying the pupillary event.^39^

Strikingly, also the vision only condition elicited a significant pupil dilation demonstrating that the visual modality alone is sufficient to reliably trigger a VER, albeit of reduced amplitude with respect to a multisensory VER. To further explore the nature of the pupillary response in vision only mice, we introduced additional variations to the protocol aiming at dissecting the specific contributions of visual processing. Specifically, we generated two versions of the original upright (UP) video to assess the effect of two distinct visual stimulations. The first variation, termed phase-scrambled (PS), involved scrambling the Fourier phase of the video frames, preserving key physical parameters, such as luminance, contrast, and motion, while disrupting the semantic integrity of the images. The second variation, referred to as inverted (IN), employed a vertical flip of the video frames, maintaining the physical properties but altering the spatial configuration. Our results revealed that the IN condition elicited a pupillary response exceeding the PS condition, although this response was significantly attenuated compared to the UP condition (Figure 3C). These findings suggest that OBS mice exhibit a greater response for social stimuli with the expected spatial configuration, implying that the pupillary response is not driven solely by alterations in physical image properties. This indicates the presence of holistic processing of mouse body configurations, similar to the mechanisms underlying facial emotional recognition in humans.^40^^,^^41^

### Brain-wide c-Fos analysis reveals a large, partially overlapping, and distributed network of activation in response to direct and vicarious emotional stimulation

**Figure 4:**
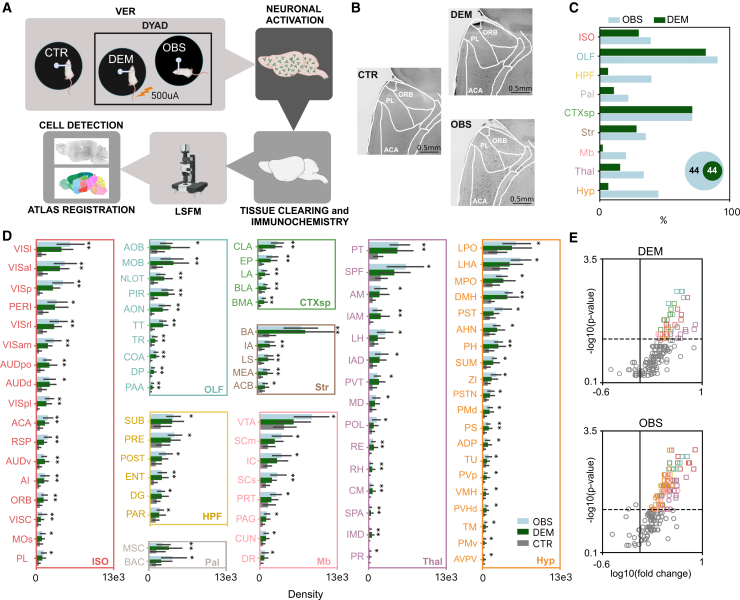
Brain-wide c-Fos mapping (A) Experimental pipeline for brain-wide c-Fos mapping in CTR, DEM, and OBS mice. (B) Representative c-Fos-immunolabeled brains in the three conditions, with magnification over prefrontal cortical regions. (C) Percentage of major brain divisions with significant c-Fos activation in OBS (light blue) and DEM (green) relative to CTR. Inset: Venn diagrams showing the number of significantly activated regions in OBS and DEM versus CTR. (D) c-Fos^+^ cell density in brain regions significantly activated in OBS or DEM compared to CTR. Values are mean ±95% confidence interval (OBS: light blue; DEM: green; CTR: gray) and grouped by major brain division (n_OBS_ = 7, n_DEM_ = 7, n_CTR_ = 6; Mann-Whitney U test, *p* < 0.05). (E) Volcano plots showing fold changes in c-Fos+ cell density for DEM (top) and OBS (bottom) relative to CTR across all areas. Colored squares mark areas significantly activated in both DEM and OBS, colored circles mark areas significantly activated only in OBS, and gray circles indicate non-significant areas. Shapes are color-coded by major brain division as in D. All brain area acronyms are listed in Table S2. ORB: orbital area, ACA: anterior cingulate area, PL: prelimbic area, OBS: observer, ISO: isocortex; OLF: olfactory areas; HPF: hippocampal formation; CTXsp: cortical subplate; Str: striatum; Pal: pallidum; Thal: thalamus; Hyp: hypothalamus; Mb: midbrain, DEM: demonstrator, CTR: control. (∗: *p* < 0.05. ∗∗: *p* < 0.01, ∗∗∗: *p* < 0.001).

To clarify the neural circuits activated during DER and VER, we conducted brain-wide analysis of c-Fos activation in the OBS and the DEM with respect to control (CTR) mice. Mice belonging to the three groups were sacrificed 90 min after the end of stimulation and c-Fos activation was assessed by brain-wide immunolabeling, iDISCO tissue clearing, and light-sheet microscopy (Figures 4A and 4B). The CTR mice were head-fixed in the same setup for the same duration of experimental mice but did not receive shocks or observe any shocked mouse. No difference in locomotor activity was present between OBS and CTR mice (OBS: 14.38 ± 5.04 SEM and CTR: 10.98 ± 4.46 SEM cm/s, *t* test *p* value = 0.55). ClearMap2 toolbox^42^ was used to align the images to the Allen Atlas Mouse Brain (25 μm–v2) and to perform automatic brain-wide detection of c-Fos^+^ cells in 169 regions across the entire brain (see STAR Methods for details). To avoid errors due to mis-alignment or tissue damage, the medulla, the pons and the cerebellum were excluded from the analysis.

We identified 88 brain regions significantly modulated in either the OBS or the DEM group compared to the CTR group (Figures 4C–4E and S4C; Table S2). These regions spanned nearly all major brain structures, with a higher proportion of recruited areas in the OBS group compared to the DEM across all primary structures except the cortical subplate (Figure 4C). Interestingly, 44 out of 88 regions showed c-Fos upregulation exclusively in the OBS group, while every region activated in the DEM group was also recruited in the OBS (Figures 4C and 4D). This pattern suggests the presence of two co-activated networks: one specific to ECo, and another shared circuit engaged in processing aversive emotional stimuli. Areas previously known to be involved in ECo,^11^^,^^27^^,^^30^ such as the anterior cingulate cortex (ACA), agranular insular area (AI), nucleus accumbens (ACB), mediodorsal thalamus (MD), lateral habenula (LH), medial amygdala (MEA), basolateral amygdala (BLA), zona incerta (ZI), ventral tegmental area (VTA), and periaqueductal gray (PAG) were significantly activated in our analysis.

Since values of c-Fos^+^ cell density provide a single activation index for each region per animal, we then performed an estimation of the interregional co-activation by calculating the Pearson correlation across subjects belonging to the same experimental group and across all pairs of brain areas. We found an overall stronger correlation in the DEM group compared to the OBS and CTR conditions (Figure S4A; median value: 0.75 for DEM, 0.4176 for OBS and 0.4385 in CTR, *p* < 0.001, Kruskal-Wallis test), indicating that, despite the recruitment of partially overlapped brain circuits, the overall network is different under the two conditions.

**Figure 5:**
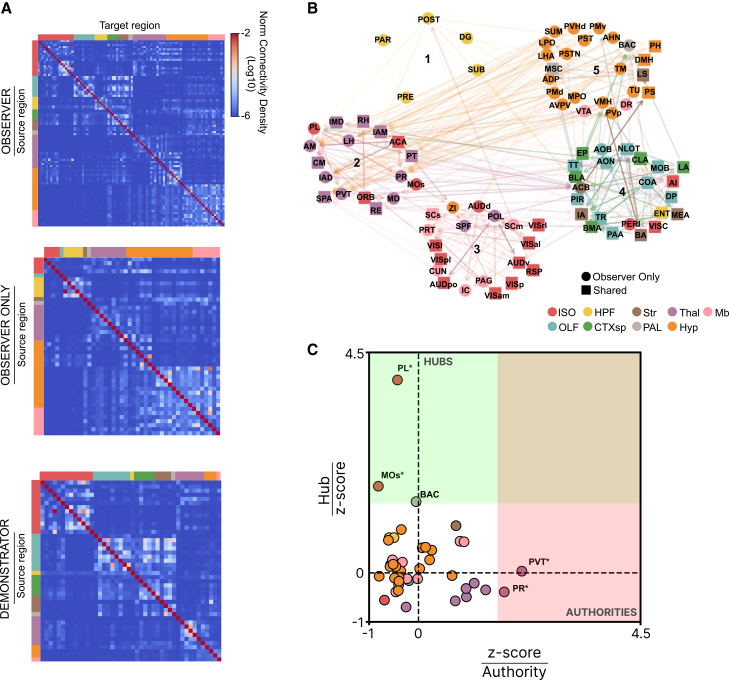
Network analysis of activated areas (A) Weighted-directed matrices of anatomical connectivity among brain areas significantly activated in OBS (top), uniquely activated in OBS (center), and activated in DEM (bottom). (B) Partitioned network of activated areas based on anatomical connectivity, displaying only the top 5% strongest connections. Line opacity reflects connection strength, arrows indicate directionality, circles represent areas activated only in OBS, squares represent areas shared between OBS and DEM, and numbers (1–5) label network partitions. (C) HITS analysis of areas activated exclusively in OBS (bootstrap procedure). Green field indicate regions with statistically significant Hub-scores (PL, MOs, and BAC; ∗: *p* < 0.05), and pink field indicate regions with statistically significant Authority-scores (PR and PVT; ∗: *p* < 0.05). Areas are color-coded according to major brain division. ISO: isocortex; OLF: olfactory areas; HPF: hippocampal formation; CTXsp: cortical subplate; Str: striatum; Pal: pallidum; Thal: thalamus; Hyp: hypothalamus; Mb: midbrain. Acronyms for all brain areas are listed in Table S2.

To investigate the anatomical connectivity patterns between the recruited brain regions, we constructed an anatomical connectome using data from the Allen Brain Connectivity Atlas^43^ generating a weighted, directed matrix representing connection strengths between the activated regions (Figure 5A). We then applied the Leiden algorithm^44^ to partition the network based on connection density. This analysis identified five clusters of interconnected regions, highlighting the organization of the network based on connection density (Figure 5B) and the complex multisensory bases of ECo. A first cluster was purely hippocampal activated only in the OBS group, a second cluster included mostly thalamic and prefrontal cortical areas; a third cluster included sensory subcortical and cortical areas; a fourth cluster included areas important for emotional control such as parts of the amygdala, the insular and visceral cortex, and olfactory areas; and a fifth cluster containing mainly hypothalamic areas.

Given that the OBS group exhibits a unique array of brain regions activated specifically during ECo, we conducted a more detailed analysis of the connectivity patterns within this circuit to better understand the role of each region in the network. Specifically, we quantified the roles of individual regions as hubs and authorities within the network using the hyperlink-induced topic search (HITS) algorithm.^45^ An authority is defined as a node that integrates a significant amount of information from other key nodes, while a hub is a node that transmits information to other nodes of the network. To calculate the significance level of each area as a hub or authority in the observed network, we compared the values to the average hub and authority scores obtained using random networks generated via bootstrapping (see STAR Methods). Figure 5C shows that the prelimbic cortex (PL), secondary motor area (MOs), and bed nucleus of the anterior commissure (BAC) emerge as key hubs, while the paraventricular nucleus of the thalamus (PVT) and perireunensis nucleus (PR) are key authorities.

### Coupled pupillary and neural responses in dyads of observer and demonstrator mice

**Figure 6:**
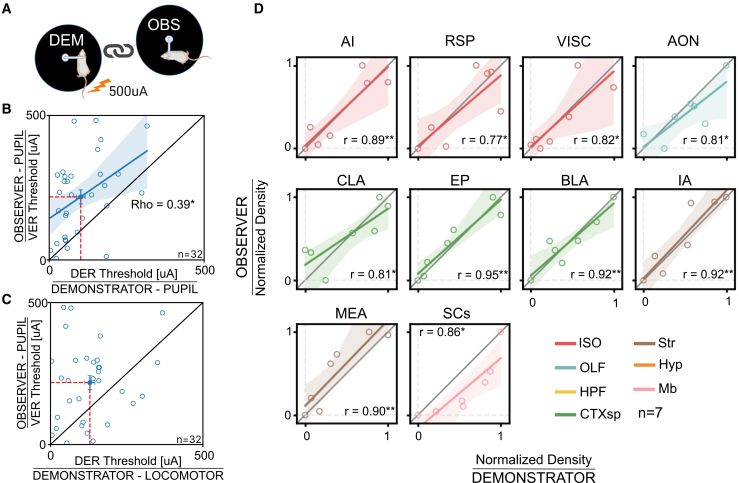
Within-dyad correlation in emotional responses and c-Fos activation (A) Sketch of a dyad. (B) Correlation between the pupillary VER thresholds of OBS and the pupillary DER thresholds of DEM in dyads. Empty circles represent values for dyads, the solid circle indicates the mean value across dyads ± SEM. Thick line indicates correlation line and shaded area is 95% CI (*n* = 32; Spearman’s ρ = 0.39, *p* < 0.05). (C) Correlation between the pupil VER thresholds of OBS and the locomotor DER thresholds of DEM in dyads. Empty circles represent values for individual dyads, the solid circle indicates the mean value across dyads ± SEM (*n* = 32; Spearman’s ρ = 0.13, *p* = 0.46). (D) Correlation between min-max scaled c-Fos activation of OBS and DEM in dyads. Only brain areas showing a significant correlation are reported. Single circles represent individual dyads, and dashed lines mark zero. Thick lines indicate correlation lines and shaded areas are 95% CI (*n* = 7; Pearson’s correlation; correlation coefficients shown in figure; see Table S2 for area nomenclature). Areas are color-coded according to major brain division: ISO: isocortex; OLF: olfactory areas; HPF: hippocampal formation; CTXsp: cortical subplate; Str: striatum; Pal: pallidum; Thal: thalamus; Hyp: hypothalamus; Mb: midbrain. (∗: *p* < 0.05. ∗∗: *p* < 0.01).

ECo is a process observed in individuals exposed to the emotions of others, leading members of a dyad to potentially exhibit similar behavioral patterns. To test this possibility, we first analyzed the correlation between the pupillary responses of the OBS during VER and of the DEM during DER within each dyad (Figure 6A). Our findings revealed a positive correlation between the pupil dilation thresholds of observers and demonstrators within each dyad (Figure 6B). This correlation was not detected when comparing pupil dilation thresholds of observers with locomotor activity thresholds of demonstrators (Figure 6C). This result suggests that the threshold for ECo of the OBS mouse is a function of the DEM distress. To study in depth this possibility we asked if the link between subjects of the same dyad could also be reflected in the activation of brain areas. We thus assessed the correlation of c-Fos^+^ cell density for all dyads, restricting the analysis to the 44 brain areas which were significantly recruited in both the OBS and DEM groups. We compared the results with a surrogate dataset generated by shuffling the animal pairs across dyads and we only considered areas showing a correlation coefficient higher than the 95th percentile of the shuffled distribution. Strikingly, a similar OBS-DEM dyad correlation was identified in the c-Fos activation. In particular, the strongest association was observed in 10 brain areas, including the MEA, BLA, the sensory related superior colliculus (SCs), and AI (Figure 6D). These regions are critical for processing emotional stimuli and regulating arousal^30^^,^^46^^,^^47^ further supporting the link between shared distress and neural activation in the dyad.

## Discussion

Our results demonstrate that pupil dynamics are a reliable and sensitive indicator of both direct and vicarious emotional reactivity to aversive stimulation. However, while both pupil dilation and locomotor activity were correlated with the intensity of the response to DER, only pupillary responses were selectively triggered during ECo. Thus, pupil dilation during ECo can reveal forms of covert responses that cannot be detected with analysis of locomotion. Importantly, pupillary DER and VER were accurately fitted by psychometric functions determining ECo sensitivity at single subject level.

It could be hypothesized that the observer’s response reflects a reaction to the salient cue of the demonstrator movement rather than to its affective state. However, we found no correlation between the demonstrator locomotor threshold and the observer pupillary VER threshold (Figure 6C). If the observer response were purely driven by a visually salient motor event, these measures should be tightly linked. While we acknowledge that salience may still play a contributing role, the absence of such a correlation suggests that the observer’s response reflects sensitivity to the demonstrator inferred internal state of distress, likely communicated via multiple sensory channels, rather than mere physical movement. This behavioral dissociation strengthens our interpretation that the underlying neural network captures a meaningful signature of ECo. Altogether, these results highlight the usefulness of pupillometry as a non-invasive, highly translational, and objective quantification of emotional responses.

Brain-wide c-Fos analysis revealed that while 44 regions were activated in demonstrators during aversive stimulation, a broader network of 88 regions was activated in the observers during ECo. While some of these areas were previously described to be involved in ECo,^27^ our analysis indicates that a relatively large network is activated in the OBS brain. Importantly, the OBS network includes all of the regions activated in the DEM, indicating an overlap between the circuits engaged in both aversive stimulation and ECo. These findings raise interesting parallels with the mirror neuron system, which has been extensively studied for its role in action recognition and imitation.^48^^,^^49^ While traditionally associated with motor functions, the mirror system has also been implicated in emotional recognition and empathy.^50^^,^^51^^,^^52^ The overlap between the regions activated during direct aversive stimulation in the DEM and ECo in the OBS suggests that ECo could involve a similar mirroring process. This mirroring could extend beyond the motor system to facilitate emotional and affective processes potentially mediated by subcortical regions, such as the amygdala and hypothalamus, which are key nodes in the regulation of emotional states.

The strong coupling between the brain activation of the OBS and the DEM is also prompted by the strong correlation between c-Fos activation in some regions of the OBS and DEM animals belonging to the same dyad. Notably, 10 specific brain regions, including the medial and basolateral amygdala, the superior colliculus, and insular cortex, showed significant correlations in activation levels between the subjects within the dyad. These regions are known to be involved in processing social and emotional stimuli, and regulating arousal,^47^^,^^53^^,^^54^ suggesting that the emotional states of the DEM and the OBS are closely aligned at neural level.

Interestingly, among these areas are structures bridging arousal and emotional processing: for example the SC shows correlated activity with pupil size,^55^ and also mediates visually induced innate fear.^56^ Another example is the BLA that integrates its role in emotional regulation with noradrenergic signals related to arousal, originating from the locus coeruleus, a nucleus known to play a key role in arousal-related pupil regulation.^16^ The activation coupling between DEM and OBS may underlie the alignment of emotional responses seen in ECo, further supporting the idea that ECo involves a shared neural substrate between individuals.^57^^,^^58^

Our analysis demonstrated that both subcortical and cortical regions associated with visual, auditory, and olfactory systems are activated by ECo. This aligns with the hypothesis that ECo is driven by multisensory integration of various sensory inputs.^27^^,^^59^^,^^60^ Pupil analysis supports this interpretation, indicating that a VER can occur without direct visual contact between the OBS and DEM, likely mediated by olfactory or auditory cues. Nevertheless, the resulting pupillary VER was reduced in magnitude. Similarly, videos of tail-shocked demonstrators elicited a diminished VER, reinforcing that complete VER expression depends on multisensory integration. Notably, the detection of the DEM aversive response was highly specific to the spatial configuration of the image and could not be replicated by videos with similar visual characteristics but altered spatial configuration. Although it is well established that mice can discriminate conspecifics based on their emotional state,^61^^,^^62^ our findings suggest that this ability may involve a specialized system for visually recognizing emotions in conspecifics.

VER elicited a broader activation of brain regions in the OBS compared to the DEM, which may subserve the processing of social and emotional signals. For example the whole hippocampal cluster was significantly activated only in observers. This suggests that the OBS brain not only mirrors the emotional state of the DEM but also engages in higher-order processes linked to ECo.^63^ The activation of this extended neural network in the OBS could be in line with psychometric analyses of pupillary responses, which revealed significantly higher thresholds for VER compared to DER. Together, these findings offer a potential neural mechanism for understanding how ECo contributes to empathy and social bonding in both animals and humans.

Our findings have significant implications for understanding social and emotional deficits in neurodevelopmental disorders, such as autism spectrum disorder (ASD) and neurodegenerative conditions like Alzheimer’s disease (AD). ECo is a fundamental component of social interaction and empathy, both of which are dysregulated in ASD, where ECo is often decreased, leading to diminished emotional resonance.^64^^,^^65^^,^^66^ In contrast, ECo is heightened in individuals with AD, possibly as a compensatory mechanism in response to cognitive decline, and it may serve as an early biomarker of the disease.^32^^,^^67^^,^^68^^,^^69^ The broader network activation observed in ECo suggests that these processes go beyond basic emotional responses. Deficits or alterations in this network may contribute to the difficulties in social communication and emotional resonance seen in ASD and AD, respectively. The identification of brain regions involved in emotional processing and ECo offers promising targets for studying how genetic mutations or neurodegenerative changes affect these circuits, which could guide interventions. Additionally, the use of pupillometry as a non-invasive marker of emotional reactivity provides opportunities for detecting subtle changes in emotional processing in patients and animal models of both ASD and AD.

In conclusion, our findings highlight that pupillary responses offer a reliable window into the shared emotional states of ECo, revealing a multisensory process at work, where ECo not only reaches across brains, but reflects shared engagement of neural systems.

### Limitations of the study

While our findings draw interesting parallels with the mirror neuron system, it is crucial to acknowledge that our c-Fos analysis, which measures population-level neural activation, does not permit conclusions about the activity of individual mirror neurons across conditions. Determining whether the same specific neurons are active during both direct aversive stimulation and VER would necessitate a within-animal, single-neuron recording approach.

A second limitation of our study concerns the sample size used for the network and correlation analyses. Caution is therefore warranted when interpreting these results, as the limited sample for this type of analysis may affect the robustness and generalizability of the findings.

## Resource availability

### Lead contact

Requests for further information and resources should be directed to and will be fulfilled by the lead contact, Raffaele M Mazziotti (raffaelemario.mazziotti@unifi.it).

### Materials availability

This study did not generate new unique reagents.

### Data and code availability

• Data: all data reported in this paper are available on Zenodo: https://doi.org/10.5281/zenodo.18031401, and an interactive web application for data exploration is accessible at raffaelemazziotti.github.io/ECo_webapp.
• Code: code and analysis pipelines are available on Zenodo: https://doi.org/10.5281/zenodo.18031401.
• Additional information: any additional information required to reanalyze the data reported in this paper is available from the lead contact upon request.

## Acknowledgments

We gratefully acknowledge NVIDIA Corporation’s support with the Jetson AGX 10.13039/100010933Xavier Developer Kit donation for this research. This work was partially supported by 10.13039/100010787AIRETT Associazione Italiana per la sindrome di Rett. Funded by the European Union- Next Generation EU, Mission 4 Component 1 CUP E53C24001460006, project TNE- NEUROBRIDGE. We would also like to thank Antonella Calvello, Vania Liverani, and Renzo Di Renzo for technical assistance.

## Author contributions

Conceptualization: R.M.M., A.V., M.C., and T.P.; methodology: R.M.M., M.C., A.V., T.P., F.C.; investigation: R.M.M., A.V., M.C., T.P., S.Z., A.S., S.B., G.S., R.N., and E.M.P.; visualization: R.M.M., M.C., S.Z., A.S., and F.C.; funding acquisition: T.P., R.M.M., S.B.; writing original draft: M.C., S.Z., A.V., T.P., S.B., A.S., R.N., E.M.P., and R.M.M.

## Declaration of interests

The authors declare that they have no competing interests.

## Declaration of generative AI and AI-assisted technologies in the writing process

During the preparation of this work, the authors used ChatGPT in order to improve readability of the manuscript. After using this tool or service, the authors reviewed and edited the content as needed and take full responsibility for the content of the publication.

## STAR★Methods

### Key resources table

**Table:** 

REAGENT or RESOURCE	SOURCE	IDENTIFIER
**Antibodies**		

Rabbit anti-*c*-Fos	Cell Signaling Technology	RRID: AB_2247211
Donkey anti-Rabbit Alexa Fluor 647	TermoFisher	RRID: AB_2536183
Donkey anti-Rabbit Alexa Fluor 647	TermoFisher	RRID: AB_2536183

**Chemicals, peptides, and recombinant proteins**		

Dibenzylether	Sigma-Aldrich	Cat#: 108014-1L
Dichloromethane	Sigma-Aldrich	Cat#: 270997-1L
Heparin	Sigma-Aldrich	Cat#: H3149
Na-Azide	Sigma-Aldrich	Cat#: S2002
Dichloromethane	Sigma-Aldrich	Cat#: 270997-1L
Heparin	Sigma-Aldrich	Cat#: H3149
Na-Azide	Sigma-Aldrich	Cat#: S2002

**Deposited data**		

Allen Brain Connectivity Atlas	Knox et al.^43^	https://github.com/AllenInstitute/mouse_connectivity_models
Interactive web app for data exploration	This paper	raffaelemazziotti.github.io/ECowebapp
Full code and analysis pipelines	This paper	Zenodo: https://doi.org/10.5281/zenodo.18031401

**Experimental models: Organisms/strains**		

Mouse: C57BL6J wild-type (M/F; P60-P120)	Charles River	RRID:IMSR_JAX:000664

**Software and algorithms**		

BigStitcher (ImageJ plugin)	Hörl et al.^70^	https://github.com/JaneliaSciComp/BigStitcher
ClearMap2	Kirst et al.^42^	https://github.com/ClearAnatomics/ClearMap
Elastix	Klein et al.^71^; Shamonin et al.^72^	RRID:SCR_009619
igraph	Csárdi et al.^73^	RRID:SCR_019225
ImspectorPro	Miltenyi Biotec	v 7.1.4
matplotlib	https://matplotlib.org/	RRID:SCR_008624; v 3.8.2
MEYE	Mazziotti et al.^74^	https://github.com/raffaelemazziotti/MEYE
NetworkX	https://networkx.github.io/	RRID:SCR_016864
pingouin	https://pingouin-stats.org/	RRID:SCR_022261; v 0.5.5
Python	Python.org	RRID:SCR_008394; v 3.6; v 3.9.18
rpy2	https://rpy2.github.io/	RRID:SCR_024701; v 3.5.11
scipy	Virtanen et al.^75^	RRID:SCR_008058; v 1.13.1
Visual Studio Code	Microsoft	RRID:SCR_026031
ClearMap2	Kirst et al.^42^	https://github.com/ClearAnatomics/ClearMap
Elastix	Klein et al.^71^; Shamonin et al.^72^	RRID:SCR_009619
igraph	Csárdi et al.^73^	RRID:SCR_019225
ImspectorPro	Miltenyi Biotec	v 7.1.4
matplotlib	https://matplotlib.org/	RRID:SCR_008624; v 3.8.2
MEYE	Mazziotti et al.^74^	https://github.com/raffaelemazziotti/MEYE
NetworkX	https://networkx.github.io/	RRID:SCR_016864
pingouin	https://pingouin-stats.org/	RRID:SCR_022261; v 0.5.5
Python	Python.org	RRID:SCR_008394; v 3.6; v 3.9.18
rpy2	https://rpy2.github.io/	RRID:SCR_024701; v 3.5.11
scipy	Virtanen et al.^75^	RRID:SCR_008058; v 1.13.1
Visual Studio Code	Microsoft	RRID:SCR_026031

**Other**		

3D printer	BambuLab	X1C
Computer	NVIDIA	Jetson AGX Xavier
Stimulus Isolator	World Precision Instruments	A320R
Ultramicroscope II	Miltenyi Biotec	N.A.
Zyla 5.5 sCMOS Camera	Andor	RRID:SCR_021136
Computer	NVIDIA	Jetson AGX Xavier
Stimulus Isolator	World Precision Instruments	A320R
Ultramicroscope II	Miltenyi Biotec	N.A.
Zyla 5.5 sCMOS Camera	Andor	RRID:SCR_021136

### Experimental model and study participant details

#### Animals

Mice were maintained in rooms at 22°C with a standard 12-h light-dark cycle. During the light phase, a constant illumination below 40 lux from fluorescent lamps was maintained. Food (standard diet, 4RF25 GLP Certificate, Mucedola) and water were available *ad libitum* and changed weekly. Open-top cages with wooden dust-free bedding were used. All the experiments were carried out according to the directives of the European Community Council (2011/63/EU) and approved by the Italian Ministry of Health (aut n. 357/2024-PR; prot. B4BB8.51). All necessary efforts were made to minimize both stress and the number of animals used. Weaning was performed on postnatal day (P)21-23. We tested male and female wild-type C57BL/6J (from P60 to P120; Charles River), and data were analyzed separately by sex. No significant sex-dependent differences were observed (Figure S2); therefore, data were pooled across sexes for subsequent analyses. We used 32 mice (16 males, 16 females) in a longitudinal design (Figure 1A). Of the 32 mice tested in the DER and VER conditions, 20 were later included in the brain-wide c-Fos experiment and 12 in the video-only condition. Additional 6 mice were added to the video-only condition to increase sample size. Video-only assessment was preceded by the same protocol used for the other mice. Before the experiments, mice were handled for 1 week for 5 minutes each day; after, they were gradually introduced to head-fixation and to wearing a stimulation electrode at the base of the tail, for an increasing amount of time for at least 5 days. To ensure familiarity with the experimental settings, mice were habituated in two different contexts: Context 1 consisted of the head-fixation apparatus with a curved monitor (24-inch Samsung CF390) placed 13 cm in front of the mouse, displaying a uniform gray background (mean luminance: 8.5 cd/m^2^). Context 2 was a different enclosure designed to accommodate two head-fixation setups. The habituation schedule was as follows: on Days 1-2, animals underwent two daily sessions of 10 minutes each, one in Context 1 and the other in Context 2, while alone. On Day 3, the same procedure was applied but with 20-minute sessions. On Day 4, animals received one 40-minute session in Context 1, paired with a conspecific. On Day 5, they received one 60-minute session in Context 2, again in pairs. To prevent stress transmission to cagemates, reduce social buffering effects, and allow habituation to isolation, mice were single housed for 1 hour at the end of each session.^76^^,^^77^^,^^78^^,^^79^

### Method details

#### Surgery

Mice were deeply anesthetized using isoflurane (3% induction and 1.5% maintenance), placed on a stereotaxic frame and head-fixed using ear bars. Prilocaine was used as a local anesthetic for the acoustic meatus. Body temperature was maintained at 37°C using a heating pad. The eyes were treated with a dexamethasone-based ophthalmic ointment (Tobradex, Alcon Novartis) to prevent cataract formation and keep the cornea moist. Respiration rate and pedal reflex were checked periodically to maintain an optimal level of anesthesia. Prior to scalp removal, a subcutaneous local injection of lidocaine (2%) was performed. After scalp removal, the skull surface was carefully cleaned with saline solution. After it dried, a thin layer of cyanoacrylate was poured over the exposed bone to attach a custom-made head post that was composed of a 3D printed base equipped with a glued set screw (12 mm long, M4 thread, Thorlabs: SS4MS12). The implant was secured to the skull using cyanoacrylate and ultraviolet curing dental cement (Fill Dent, Bludental). At the end of the surgical procedure, mice were left in a heated cage for recovery. After 1 h, mice were returned to their home cage. As antalgic therapy, paracetamol (100 mg/kg) was dissolved in drinking water for 3 days. At least 7 days were provided for the animals to fully recover before beginning of habituation procedures.

#### Pupillometry and locomotor activity recordings

For pupillary and locomotor activity recordings, mice were head-fixed and free to run on a circular treadmill. A modified version of the apparatus proposed by Silasi et al.^80^ equipped with a 3D printed circular treadmill (⌀ 18 cm) was employed. Locomotor activity was detected using a rotary encoder (E38S6G5-600B-G24N). A USB camera (oCam-5CRO-U, Withrobot Lens: M12 25 mm) connected to a Jetson AGX Xavier Developer Kit (NVIDIA) running a custom Python script (30fps) was used to record the pupil. Real-time pupillometry was performed using MEYE.^74^ To ensure a uniform light on the pupil an infrared (IR) illuminator was used. Pupillary and locomotor time series were recorded at 20 Hz. Trials with saccades, eye closures or transient detection failures were excluded from further analysis. In the pupillary trials, blinking activity was removed by applying a median filter to the absolute value of the first derivative of the pupil track, normalized from 0 to 1. Derivative values exceeding 0.2 were replaced with NaN and then linearly interpolated. The pupil track was smoothed using Savitzky-Golay filtering with a polynomial order of 6 and a window length of 1.5 seconds.

#### Direct emotional response assessment

To assess pupillary and locomotor responses to aversive stimulation via the delivery of tail shocks, head-fixed mice were placed in Context1. A stimulation electrode was attached to the base of the mouse tail and plugged to a stimulus isolator (World Precision Instruments, A320R). Pupillary area and locomotor activity were continuously recorded during the experiment. An ascending and then descending ladder of electric stimuli (0 μA, 50 μA, 150 μA, 300 μA, 500 μA, 0 μA, 500 μA, 300 μA, 150 μA, 50 μA; duration of 0.2 sec, interstimulus time of 60 sec) was delivered to mice. The ascending-descending ladder was repeated 3 times with a total of 6 trials for each stimulation level. The total duration of the experiment was 30-35 min.

#### Vicarious emotional response assessment

In this experiment, same-sex cagemate mice dyads were used. One mouse was designated as the OBS and the other as the DEM. The experiment was conducted in Context 2, 48 hours after the DER test. Roles were assigned in a rolling dyad design, where the OBS mouse in one pair served as the DEM in the next pair. Because the first DEM and the last OBS from the same cage cannot follow this sequence, they were tested 48 hours later with their roles reversed (Figure S3A). Including or excluding these 6 mice undergoing VER after 48 hrs did not change the VER threshold (Figure S3D). A stimulation electrode was attached to the tail of both animals, but only the electrode of the DEM was connected to the stimulus isolator. The pupillary size of the OBS and the locomotor activity of both mice were continuously recorded during the experiment. There was no difference in locomotor activity thresholds when an animal experienced a DER and when it served as the DEM for the VER study (Figure S3B). The DEM mouse was placed at the same height but perpendicular to the line of sight of the OBS mouse, with its face centered in the binocular visual field of the OBS at a fixed distance of 13 cm (Figure 2A). An ascending and descending sequence of electric stimuli (0 μA, 50 μA, 150 μA, 300 μA, 500 μA, 0 μA, 500 μA, 300 μA, 150 μA, 50 μA; 1-second duration, with 60 seconds between stimuli) was delivered to the DEM. This sequence was repeated 6 times, resulting in 12 trials for each stimulation level. The total duration of the experiment was 1 hour.

#### VER assessment under visual occlusion

At the end of the VER assessment, to test whether ECo operates through non-visual sensory mechanisms, an opaque visual occluder (black coated polystyrene) was placed between the two animals. After 5 minutes of habituation, electric stimuli (0 μA, 500 μA; 1-second duration, with 60 seconds between stimuli) were delivered to the DEM. The stimulation was repeated 12 times, resulting in 12 trials for each stimulation level. The total duration of the second part of the experiment was 20 minutes.

#### Video recording

To further investigate visual cue involvement in ECo, we recorded videos of mice undergoing a simplified version of the aversive stimulation protocol used for demonstrators. These videos were then shown to OBS mice. The recordings were made with an IR USB camera connected to a Raspberry Pi 3, running a custom Python3 script with HEVC encoding in grayscale, and saved in .mp4 format at 20 fps. An IR illuminator provided adequate lighting without altering the environmental luminance at visible wavelengths. Stimulation electrodes were attached to the tail of the mouse and connected to a stimulus isolator. The camera was positioned parallel to the mouse running direction, capturing the entire body from face to tail. To standardize the videos, the mouse eye was centered in a fixed area of the frame. Before the stimulation protocol began, a 20-minute video of baseline activity was recorded. The stimulation sequence included only control (CTR) and maximal stimulation intensities (0 μA and 500 μA; 1-second duration, with 60 seconds between stimuli, the stimulation was repeated 12 times, resulting in 12 trials for each stimulation level).

#### Video administration

OBS mice were head-fixed with a mock stimulating electrode placed at the base of the tail and positioned 13 cm from the monitor in Context 1. DEM videos were aligned such that the head of the DEM was centered within the binocular visual field of the OBS. Following a 5-minute habituation period during which observers viewed an unstimulated DEM, mice were presented with randomized video sequences of the DEM receiving either 0 μA or 500 μA tail shocks (20 s pre-stimulation, 15 s post-stimulation). Videos of the DEM were presented under three conditions: upright (unaltered video), inverted (vertically flipped), and phase-scrambled (Fourier phase randomized while preserving low-level physical properties). Each stimulation intensity was presented 18 times per condition. Pupillometry was recorded from 1 second before to 4 seconds after stimulus onset. The total duration of the experiment was 66 minutes.

#### Vicarious emotional response test for c-Fos

For the c-Fos staining experiment, 20 socially reared male and female mice (previously studied, Figure 1A) were assigned to three groups: observers, demonstrators, and controls. OBS and DEM mice were head-fixed in Context 2 with electrodes attached. After 5 minutes of habituation, demonstrators received five tail shocks (500 μA, 1-second duration, with 60 seconds between stimuli). CTR mice were head-fixed in the same context for an equivalent duration but received no stimulation and observed no shocks. Following the procedure, mice were single housed to prevent social interaction and left for 90 minutes to maximize c-Fos expression,^81^ after which they were transcardially perfused.

#### Brain-wide c-Fos immunolabeling and tissue clearing

To evaluate brain-wide c-Fos expression mice were sacrificed 90 minutes after VER, DER or CTR by transcardial perfusion with 0.09% saline solution followed by 4% paraformaldehyde (PFA) for tissue fixation. Brains were extracted, post-fixed for 3 hours in 4% PFA at RT, washed three times in 1M Phosphate Buffer Solution (PBS) for one hour, and stored in PBS Na-Azide 0.02% solution at 4°C. iDISCO protocol^82^^,^^83^ was used for brain-wide c-Fos immunolabeling and tissue clearing. The two brain hemispheres were first split and only the right hemisphere underwent the procedures. Considering this, subtle effects in midline structures may have been underestimated. However, regions bordering the midline were preserved in our analyses. Samples were dehydrated in a methanol (MeOH) gradient: 20% MeOH, 40% MeOH, 60% MeOH, 80% MeOH, 100% MeOH, 100% MeOH in MilliQ (H_2_O) for 1h each at RT on a rotating wheel, before overnight delipidation in a solution of 33% MeOH / 66% dichloromethane (DCM, Sigma-Aldrich) at 4°C. Next, samples were rinsed twice in 100% MeOH for 1h, and bleached in 5% H_2_O_2_ in MeOH at 4°C overnight. Finally, samples were washed in 100% MeOH for 1h and gradually rehydrated in 80% MeOH, 60% MeOH, 40% MeOH, 20% MeOH (1h each, in MilliQ H_2_O) and PBS (twice for 1h). Samples were then incubated on an adjustable rotator at RT in a permeabilization solution of PBS containing 0.02% Na-Azide, 0.2% TritonX-100, 20% DMSO, 0.3M glycine for 2 days followed by incubation at RT in a blocking solution of PBS containing 0.02% Na-Azide, 0.2% TritonX-100, 10% DMSO, 6% Donkey Serum, for additional 2 days. Samples were then incubated in the primary antibody solution (Rabbit anti-c-Fos antibody, 108 ug/ml, Cell Signalling, in PBS 0.02% Na-Azide, 0.2%, Tween, 10ug/mL Heparin, 5% DMSO and 3% Donkey Serum solution) on a rotating wheel at 37°C for 2 weeks. After primary antibody incubation brains were washed 6 times with a PBS solution containing 0.02% Na-Azide, 0.2% Tween, 10ug/mL Heparin. Next, samples were incubated with the secondary antibody (Donkey anti-Rabbit AlexaFluor 647, 1.5 ug/ml), in the same solution used for primary antibody, and placed at 37°C in rotation for 1 week. For tissue clearing, samples were dehydrated in a methanol gradient: 20% MeOH, 40% MeOH, 60% MeOH, 80% MeOH, 100% MeOH, 100% MeOH in MilliQ (H_2_O) for 1h each at RT on a rotating wheel and protected from the light. Delipidation was achieved with 3 hours incubation at RT in 33% MeOH / 66% DCM, followed by two washes of 20 minutes with 100% DCM. Samples were cleared in dibenzylether (DBE, Sigma-Aldrich) and protected from the light. Samples were stored in glass tubes in the dark at RT until imaging.

#### Light-sheet fluorescence microscopy (LSFM)

3D imaging of clarified samples was performed on the Ultramicroscope II (Miltenyi Biotec) equipped with a 4×/0.3NA objective and an Andor Zyla 5.5 sCMOS Camera. Samples were placed in an imaging chamber made of 100% quartz filled with DBE and illuminated from the side by the laser light. The light sheet was generated by a laser (wavelength 639 nm) and two cylindrical lenses, and the emitted signal was first filtered with a bandpass filter emission wavelength 680 nm. Laser power was set at 45% and the camera exposure time at 200.00 ms. ImspectorPro v7_1_4 software (Miltenyi Biotec) was used for image acquisition. The z-step between each image was fixed at 5 μm, and for tile imaging the overlap was set to 20%. Acquired volumes (16-bit tiff) had a radial resolution of 1.625 μm, and an axial resolution of 6.72 μm (NA = 0.035). The resulting sequences of tiff files were processed with BigStitcher software^70^ to obtain a single stitched file. Stitched files were then imported into ClearMap2 toolbox^42^ to automate the detection of c-Fos^+^ cells.

### Quantification and statistical analysis

All statistical analyses, graphing, and data processing were carried out using Python-based workflows. Light-sheet fluorescence microscopy data were analyzed with custom Jupyter notebooks executed in Visual Studio Code (version 1.104.1) using Python 3.6 and Jupyter Notebook 6.4. Pupillometry data were analyzed in JetBrains DataSpell (version 2024.2.2) with Python 3.9.18, employing matplotlib 3.8.2, pingouin 0.5.5, scipy 1.13.1, notebook 7.4.5, and rpy2 3.5.11. All the details about statistical analysis are available in Table S1.

#### Statistical analysis for pupillometry and locomotor activity

Pupillary responses to each stimulation intensity were z-scored relative to a 1 seconds prestimulus window and baseline-corrected by subtracting the responses to the 0 μA shock condition. Locomotor activity was similarly smoothed using Savitzky-Golay filtering with the same polynomial order and window length. Responses were baseline-corrected using the average value from a 1-second prestimulus window, followed by subtracting the responses to the 0 μA shock condition. The value of both pupillary and locomotor responses was defined as the average value of the signal in the interval between 1 and 2 seconds after stimulus onset. To evaluate the pupillary responses curves, we utilized a custom psychometric fitting method. This method involves fitting a sigmoid function to the data, to model the relationship between stimulus intensity and the pupillary response. The fitting process was performed with Python using nonlinear least squares optimization provided by the scipy library. The psychometric fit was performed in the following manner: the array of responses for each animal was averaged over the time period from stimulus onset (0 to 4 seconds). The arrays corresponding to the stimulus intensities were normalized, and the resulting pupillary responses were scaled between 0 and 1 for each animal. The fit is performed using the sigmoid function $f\left(x\right)=b+\frac{L}{1+e^{-k\left(x-x_{0}\right)}}$ where x stimulus intensity, x_0_ is the threshold, k is the slope of the curve, L is the upper asymptote, b is the baseline level of the curve. The threshold was defined as the stimulus intensity at which the pupillary response reaches its inflection point. The goodness of fit was evaluated using the coefficient R^2^, which measures how well the fitted curve captures the variance in the data. Statistical analyses were run in Python with Pingouin^84^ and SciPy,^75^ and Bayes factors were computed in R (4.3.1) with BayesFactor via rpy2. Assumptions were tested with Shapiro–Wilk (pingouin.normality) and homogeneity with Levene (pingouin.homoscedasticity) tests. When assumptions were met, we used one-way ANOVA for between-subjects (pingouin.anova), repeated-measures ANOVA for within-subjects (pingouin.rm_anova), and pingouin.ttest for two-level factors (paired or independent; with Welch correction when variances were unequal). When assumptions were violated, we used Friedman for within designs (pingouin.friedman) with Wilcoxon signed-rank post hoc (pingouin.wilcoxon), and Kruskal–Wallis for between designs (pingouin.kruskal) with Mann–Whitney U post hoc (pingouin.mwu). Post hoc comparisons were performed with pingouin.pairwise_tests, setting as appropriate and adjusted with Benjamini–Hochberg correction for multiple comparison.^85^ Spearman correlations were computed with scipy.stats.spearmanr. All tests were two-sided with α = 0.05.

#### Automated c-Fos+ cell detection and statistical analysis of area activation

Single stitched files (1 file/animal) were imported in ClearMap2^42^ for atlas registration and cell detection. Images were first converted into npy format tridimensional matrix, (reporting pixel intensity on x and y and stacks on the z dimension) and then aligned to the Allen Brain Atlas. For atlas registration images were down-sampled to match raw data resolution (X: 1.625 μm, Y: 1.625 μm, Z: 5 μm) with the one of the reference atlas (X: 25 μm, Y: 25 μm, Z: 25 μm). Resampled data were aligned with the Allen Brain Atlas firstly through a linear (affine) transformation, and then optimized with a second non-linear (b-spline) transformation, using the Elastix software package.^71^^,^^72^ Next, cell detection was performed in four consecutive steps: 1) Background Removal; 2) Spot/Maxima Detection; 3) Cell Shape Detection (set to match the overall number of detected cells across animals); 4) Cell Size Detection (minimum pixel size selected: 7 pixel). Cell detection outputs include the number of detected cells, their spatial coordinates, and their fluorescence intensity. Since the registration of images to the Allen Mouse Brain Atlas can be executed across multiple hierarchical levels, the identification of activated cells can be performed within brain region, area, or subarea (including cortical layers). In this study, we selected 198 brain areas from the Allen Brain Atlas hierarchy (structure level = 8), excluding brain areas associated with the Pons, Medulla, Cerebellar cortex, and Cerebellar nuclei macro-areas. To identify brain areas differentially activated across conditions, we analyzed the density of detected cells (i.e., the raw count normalized by the brain area volume, as estimated by the Allen Brain Atlas). To compare two conditions, we employed the non-parametric Mann-Whitney U test (alpha = 0.05).

Since values of c-Fos expression density provide a single activation index for each region per animal, interregional co-activation estimation was performed by calculating the Pearson correlation across subjects, rather than within subjects, across all pairs of brain areas. Next, co-activation matrices were created cross-correlating regional c-Fos expression densities across the CTR, DEM, and OBS groups. Statistically significant differences between conditions were assessed using the non-parametric Kruskal-Wallis test (alpha = 0.05). We did not correct for multiple comparisons due to the small sample size, since over-correction would lower power and increase Type II errors. This approach is consistent with recommendations for exploratory studies.^86^^,^^87^ Additionally, our use of non-parametric tests provides some protection against false positives.^88^

#### c-Fos activation in dyads

To assess the relationship between c-Fos activation in paired animals from the same dyad, we evaluated the linear correlation coefficient between paired animals in individual brain areas. We first paired animals based on the behavioral paradigm used to map brain activation, so that each OBS was paired with the corresponding DEM used during VER. We then selected the 44 brain regions that showed a significant increase in c-Fos cell density in both the OBS and DEM groups compared to the CTR. For each selected region, we evaluated the correlation coefficient between paired animals of the same dyad and compared the results with surrogate data generated by 42 different combinations of randomly shuffled paired animals across dyads. We set the threshold for significance at the 95th percentile of the distribution of shuffled data, and regions were considered to be significantly positively correlated only if they had a correlation coefficient greater than the threshold. For visualization, the density values for each area in the correlation plots were normalized using min-max scaling, calculated as (x - min(x)) / (max(x) - min(x)), which rescales all values to the range 0-1.

#### Anatomical network analysis

Brain areas with significant activation with respect to CTR were used to construct the anatomical connectome network. The normalized connection density was obtained from a publicly available dataset of data driven high resolution mouse connectome.^43^ A weighted directed graph with all the activated areas was created using the NetworkX Python library. Leiden algorithm was calculated in iGraph^73^ using the *leidenalg* Python library (https://github.com/vtraag/leidenalg) to find the partitions of the network based on the density of weighted directed connections.^44^ The Hyperlink-Induced Topic Search (HITS) algorithm was performed only in the areas activated in the OBS. The HITS algorithm is primarily used for ranking web pages but can be applied to various networks to identify important nodes. The algorithm iteratively updates these scores to reflect the mutual reinforcement between hubs and authorities, providing insight into the network structure. Areas with high hub scores might represent regions that influence many other areas, acting as connectors or broadcasters of neural information. Conversely, areas with high authority scores may signify regions that are crucial recipients or integrators of information.^89^ To evaluate the significance of hub and authority scores for specific brain areas, we implemented a bootstrap-based randomization procedure. Random networks were generated to create a null model for statistical comparison. At each iteration, a random network was generated by randomly selecting brain areas, preserving the total number of areas in the network. The target area of interest was included in every iteration, while the remaining nodes were randomly sampled from the set of brain areas analyzed using the Allen Brain Atlas. Connections from and to the same brain structure were removed. For each target brain area, 1,000 random networks were generated. In each iteration, hub and authority scores for the target area were calculated using the HITS algorithm. This process resulted in a distribution of random hub and authority scores for the target area, representing the expected scores under random conditions. We calculated the z-score to measure how many standard deviations the observed hub or authority score deviated from the mean of the bootstrap-generated distribution. The Z-score was then compared with the cumulative distribution function of the standard normal distribution to compute a one-tailed p-value.

## Supplemental information

Document S1. Figures S1–S4

Table S1. Statistical table, related to all Figures

Table S2. List of abbreviations of anatomical areas, related to Figures 4, 5, and 6
