# Supplementary material for: Pupillometry and brain-wide c-Fos mapping uncover multimodal mirror emotional contagion related networks of mice
Source: iScience. 2026 Jan 29;29(2):114827. doi: 10.1016/j.isci.2026.114827 (PMC12915283; doi:10.1016/j.isci.2026.114827)
Supplement: Document S1. Figures S1–S4 [file mmc1.pdf]

iScience, Volume *29*

Supplemental information

Pupillometry and brain-wide c-Fos mapping uncover multimodal mirror emotional contagion related networks of mice

Matteo Caldarelli, Stefano Zucca, Aurelia Viglione, Alessandra Stella, Rida Nisar, Giulia Sagona, Ester M. Papini, Fabio Carrara, Serena Bovetti, Raffaele M. Mazziotti, and Tommaso Pizzorusso

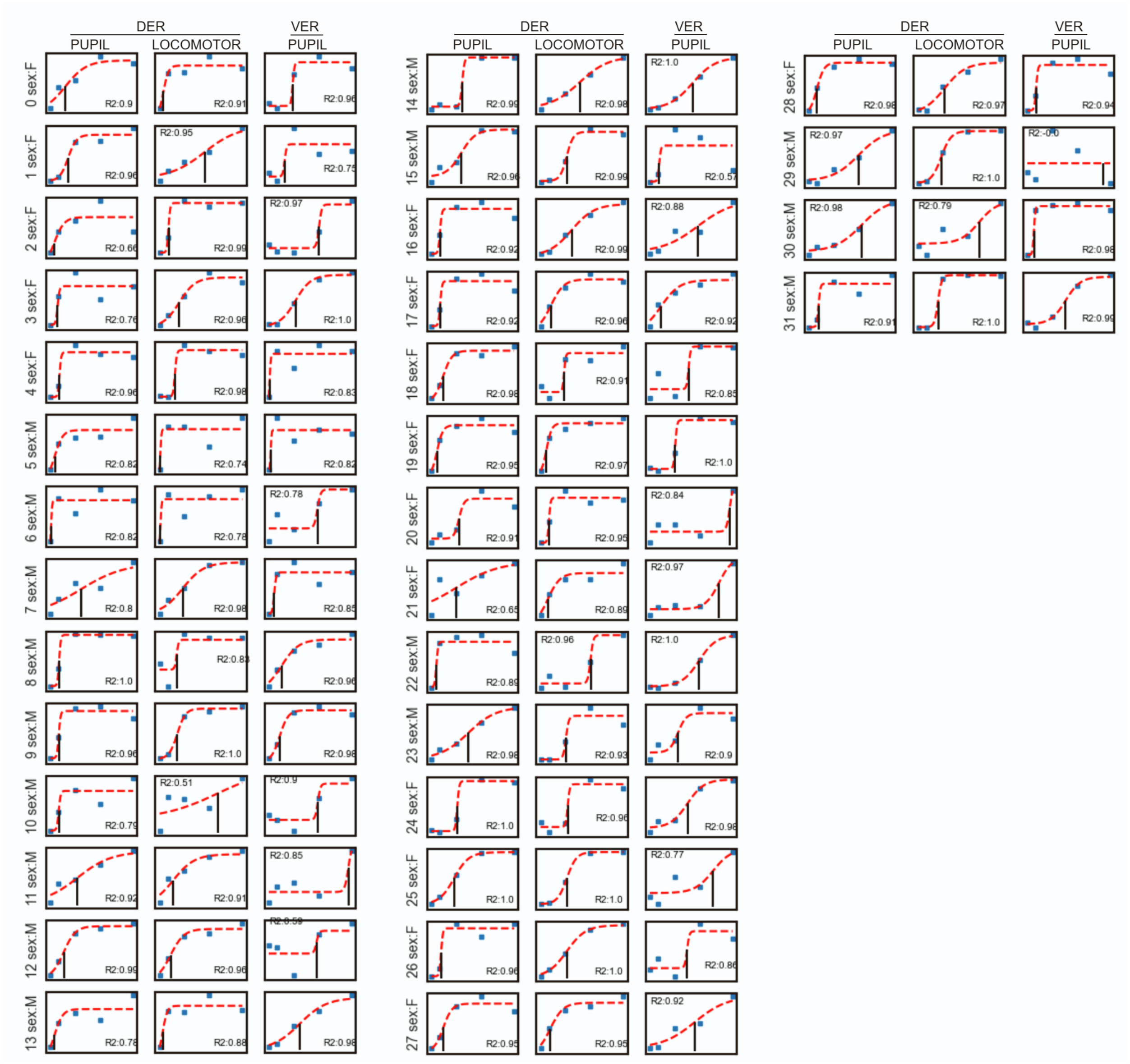

Figure S1. Individual psychometric fits for pupillary and locomotor responses. *Psychometric curves for pupillary and locomotor responses during DER, and pupillary responses during VER, are shown for every single animal. Average responses to each level (solid blue squares) were fitted with a sigmoidal function (dashed red line), and the intensity corresponding to 50% of the maximal response was defined as the response threshold (vertical black line). The goodness of fit for each curve is indicated by the R^2 value displayed. The identity of each mouse is indicated on the left of each series of graphs, with a number representing the animal and a letter (M or F) indicating sex. (n = 32)*

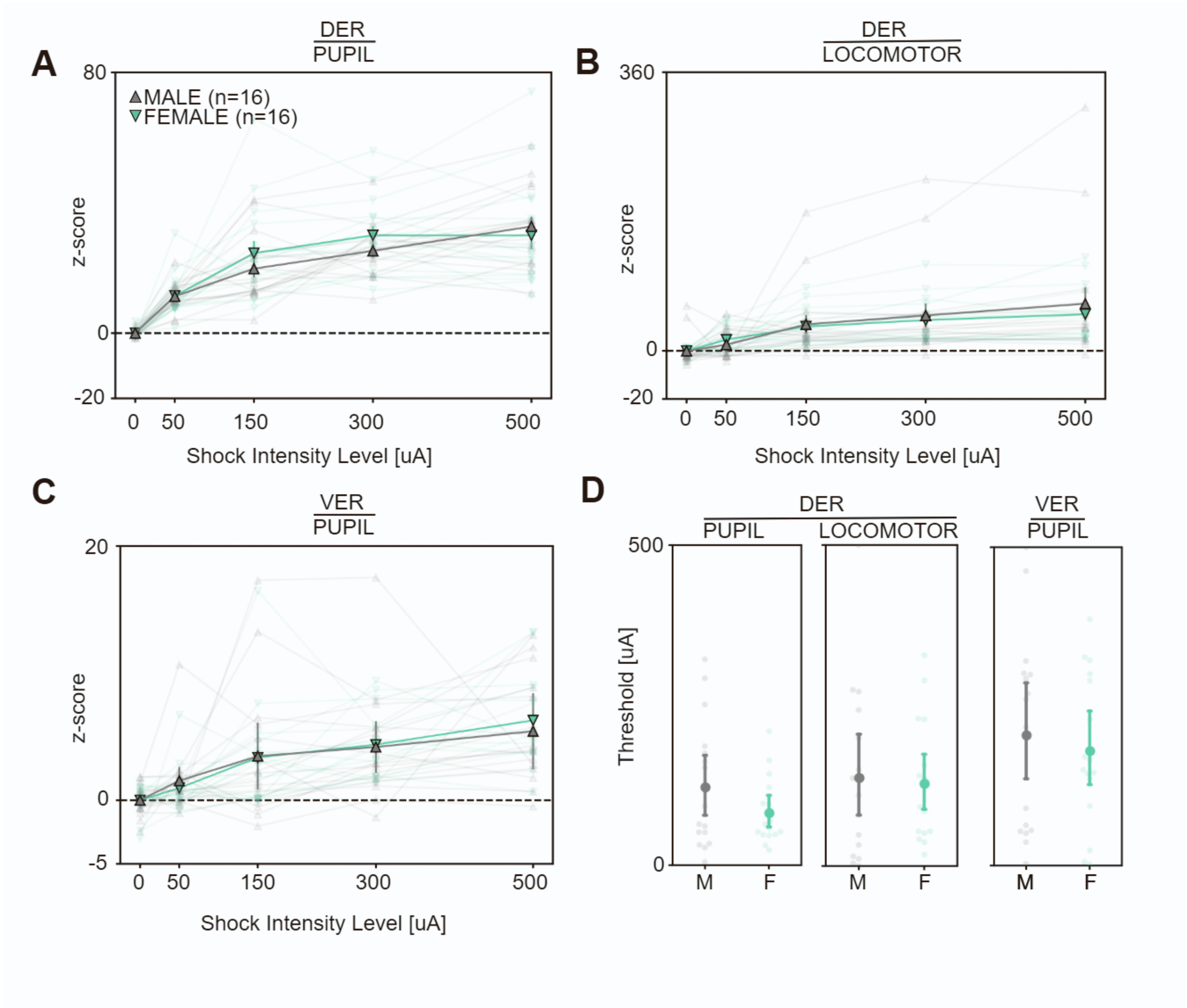

Figure S2. Sex differences in direct and vicarious emotional responses in mice. A) *Average pupillary dilation as a function of shock amplitude. Thin lines represent individual mouse averages, and thick lines indicate the mean response across all animals (n_{male} = 16, n_{female} = 16; repeated-measures two-way ANOVA, p = 0.57).* ***B)*** *Average locomotor activity as a function of shock amplitude. Thin lines represent individual mouse averages, and thick lines indicate the mean response across all animals (n_{male} = 16, n_{female} = 16; repeated-measures two-way ANOVA, p = 0.77).* ***C)*** *Average pupillary dilation in the OBS as a function of the shock amplitude applied to DEM. Thin lines represent individual mouse averages, and thick lines indicate the mean response across all animals (n_{male} = 16, n_{female} = 16; repeated-measures two-way ANOVA, $F_{1,30}$ = 0.01, p = 0.92).* ***D)*** *Comparison of response thresholds between VER (pupil) and DER (pupil and locomotor activity) conditions in male and female mice. (n_{male} = 16, n_{female} = 16; DER_{pupil}: two-sided t-test, t(30) = 1.4, p = 0.17. $DER_{locomotor}$: two-sided t-test, t(30) = 0.22, p = 0.82. VER_{pupil}: two-sided t-test, t(30) = 0.48, p = 0.63).*

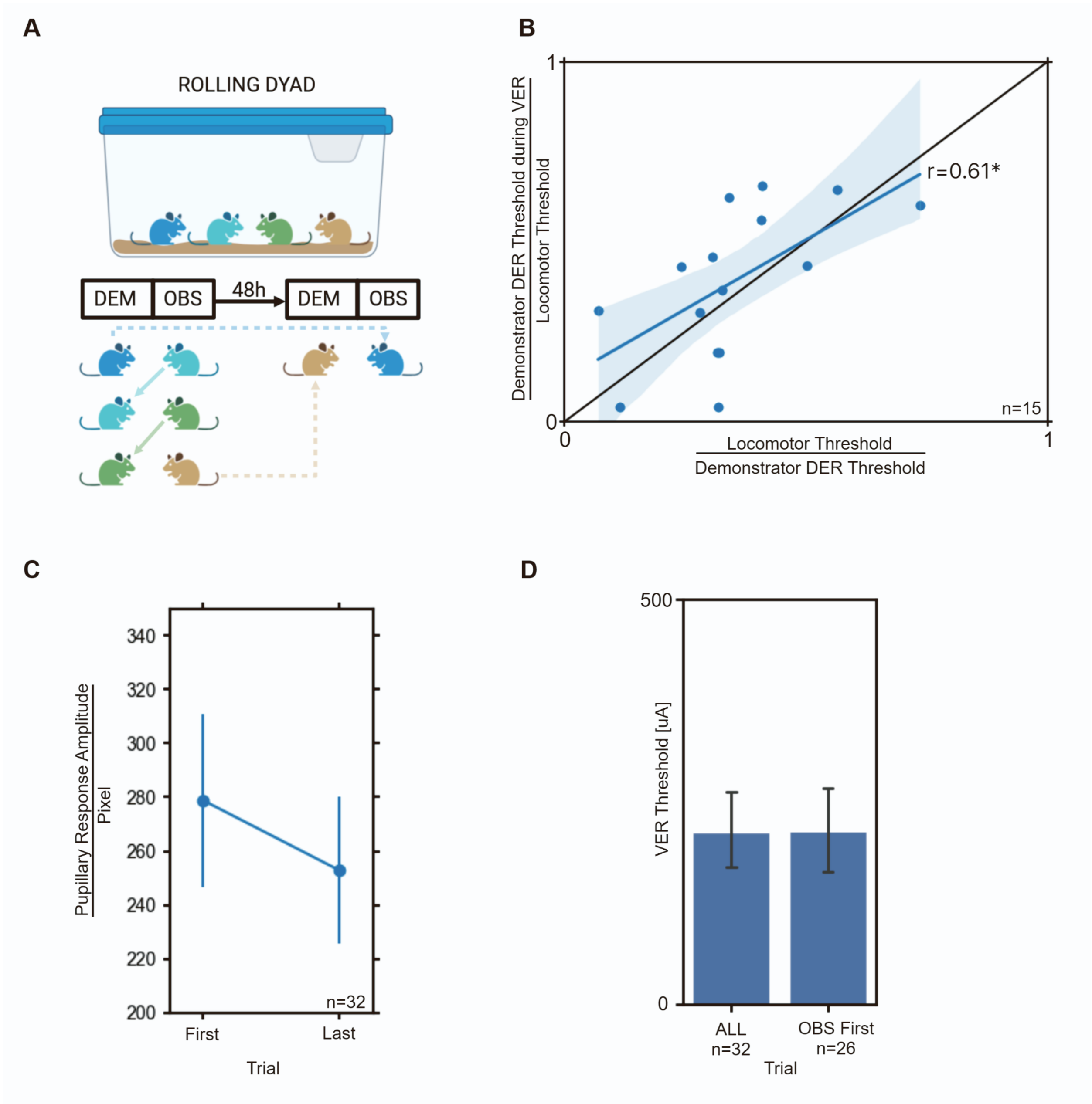

Figure S3. Rolling dyads design and effects of role reversal. A) *Rolling dyad design. All animals are cagemates and sex-matched. The first OBS of the cage serves as the DEM for the subsequent OBS. The first DEM of the cage and the last OBS of the same cage are tested with their roles reversed 48 h later.* ***B)*** *Correlation between DEM locomotor response thresholds during VER and DER conditions (n = 15; Spearman's ρ = 0.61, p = 0.02, BF_{10} = 4.593).* ***C)*** *Comparison of pupillary response raw amplitude during the first vs. last trial of DER (n = 32; two-sided t-Test, t(31) = 1.02, p = 0.31, BF_{10} = 0.306).* ***D)*** *Comparison of VER pupillary thresholds of all OBS, including mice that were first DEM and then became OBS 48 h later (rolling dyad) vs. mice that served as OBS before becoming DEM (t(54.1) = 0.01; two-sided t-test; 95% CI [-0.15, 0.15]; Cohen's d = 0; p = 0.99; BF_{10} = 0.267; power = 0.05; n_{ALL} = 32; $n_{OBSfirst}$ = 26).*

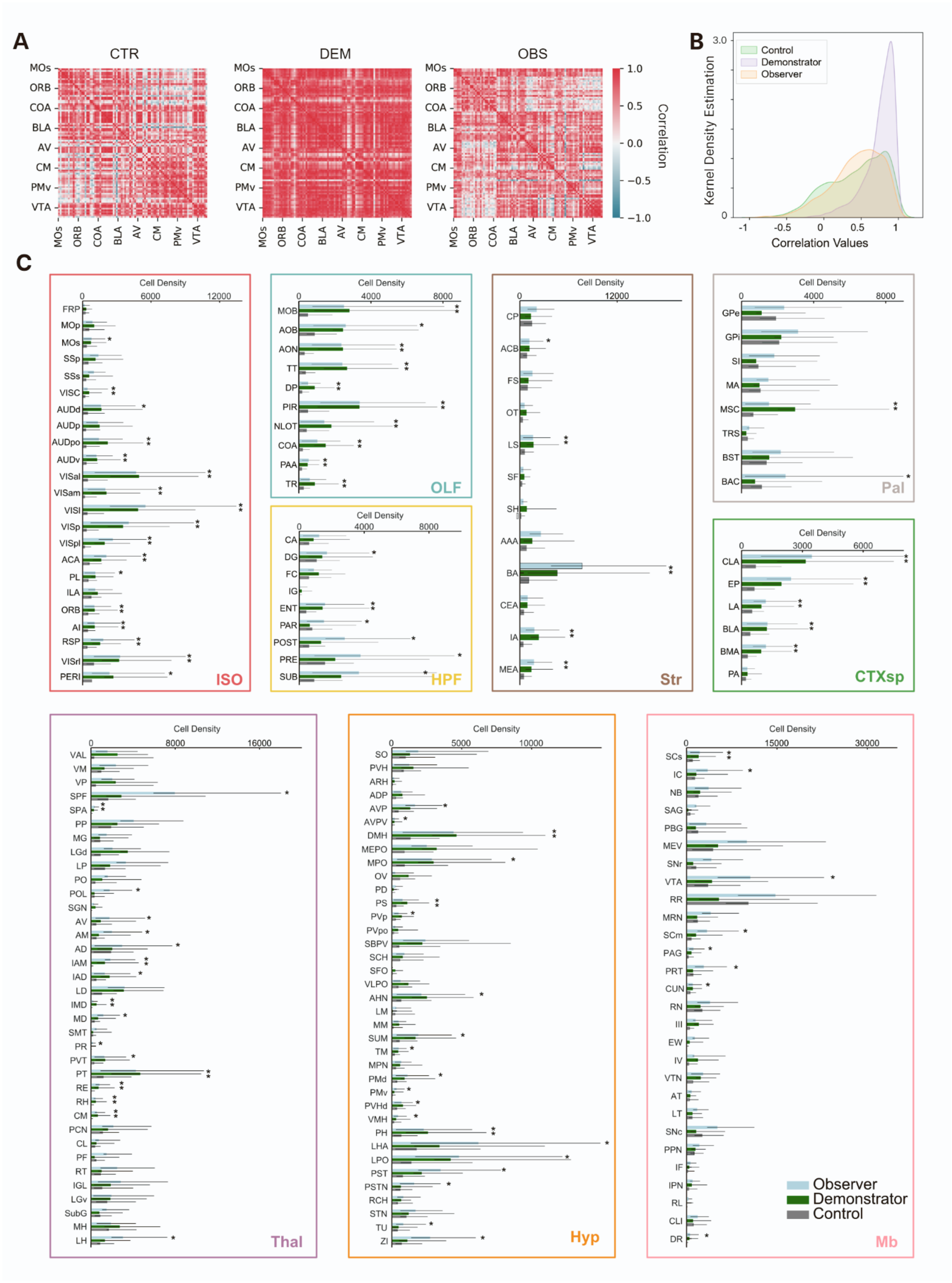

Figure S4. c-Fos expression across experimental groups. A) *Pairwise correlation of c-Fos⁺ cell*

density between all brain regions across animals from the CTR (left), DEM (center), and OBS (right) groups. ***B)*** *Probability density distribution of calculated correlation coefficients in the three experimental conditions.* ***C)*** *Bar graphs showing c-Fos*$^{+}$ *cell density for each brain area in the three experimental conditions (OBS, light blue; DEM, green; CTR, gray). Regions are grouped based on the major brain division of the Allen Brain Atlas hierarchy: ISO: isocortex; OLF: olfactory areas; HPF: hippocampal formation; CTXsp: cortical subplate; Str: striatum; Pal: pallidum; Thal: thalamus; Hyp: hypothalamus; Mb: midbrain. Asterisks indicate a significant difference between the OBS or DEM (based on the position over the single bar) compared with the CTR group (t-test; exact p-values reported in Table 1). ORB: orbital area; ACA: anterior cingulate area; PL: prelimbic area).*
